# Segmental maternal uniparental disomy of chromosome 7q in a patient with congenital chloride diarrhea

**DOI:** 10.1002/jcla.23862

**Published:** 2021-06-04

**Authors:** Juanjuan Lyu, Zhuo Huang, Hongbo Chen, Xiaomei Sun, Ying Liu, Chuanjie Yuan, Li Ye, Dan Yu, Jin Wu

**Affiliations:** ^1^ Department of Pediatrics West China Second University Hospital Sichuan University Chengdu China; ^2^ Key Laboratory of Birth Defects and Related Diseases of Women and Children Ministry of Education West China Second University Hospital Sichuan University Chengdu China

**Keywords:** Congenital chloride diarrhea, mUPD, Silver–Russell syndrome, *SLC26A3*

## Abstract

**Background:**

The main symptoms of congenital chloride diarrhea (CCD) main symptoms are watery diarrhea, hypochloremia, and hypokalemic metabolic alkalosis. Silver–Russell syndrome (SRS) is a heterogeneous imprinting disorder characterized by severe intrauterine retardation, poor postnatal growth, and facial dysmorphism.

**Methods:**

Parent‐offspring trio whole‐exome sequencing was used to identify the causal variants. Sequencing reads were mapped to the reference of human genome version hg19. Sanger sequencing was performed as a confirmatory experiment.

**Results:**

The proband was a patient with SRS caused by maternal uniparental disomy 7. The CCD of the proband was caused by homozygous variant c.1515–1 (IVS13) G>A; both mutated alleles were inherited from her mother.

**Conclusion:**

We report the first clinical case of CCD and SRS occurring together. Patients with milder phenotypes may be difficult to diagnose in early stage, but close monitoring of potential complications is important for identification.

## Graphical abstract

We report the first case of a female child presenting with CCD accompanied by maternal segmental UPD of chromosome 7 confirmed by molecular diagnosis. The proband was the only patient in the family and harbored a SLC26A3 variant (NM_000111.3:c.1515‐1G>A). The mother of the patient was heterozygous, whereas the father was wild‐type. After treatment of CCD, the patient still failed to thrive and had a typical dysmorphic feature. In the second genetic analysis, we found that at least 86.51 Mb of genome 7q11q36 (chr7: 65446986–151960086) was maternal uniparental disomy. We revised the diagnosis to CCD combined with SRS.

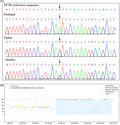

## 1 INTRODUCTION

Congenital chloride diarrhea (CCD, OMIM 214700), caused by loss‐of‐function mutations of the *SLC26A3* gene, is a rare congenital autosomal recessive disorder, characterized by antenatal polyhydramnios, premature birth, postnatal dehydration, failure to thrive in the setting of hypokalemic metabolic alkalosis, and high fecal chloride.^1^ More than 250 cases have been reported since the disorder was first published in two simultaneous cases by Darrow and Gamble in 1945.

Silver–Russell syndrome (SRS, OMIM 180860) was first described in 1953 and 1954, and is an epigenetic disorder characterized by small for gestational age (SGA) and postnatal growth failure, relative macrocephaly at birth, triangular facial appearance with protruding forehead, body asymmetry, feeding difficulties, and less common features.^2^ The most common genetic abnormalities of SRS are demethylation on chromosome 11p15 (11p15 LOM) and maternal uniparental disomy (UPD) of chromosome 7 (UPD(7)mat), sometimes restricted to part of 7q, which accounts for 30%–60% and 5%–10% of cases, respectively.^2^ UPD(7)mat was detected as the first major molecular alteration in SRS; however, the clinical manifestations are less typical than those of 11p15 LOM.^3^

Here, we report the first case of a female child presenting with CCD accompanied by maternal segmental UPD of chromosome 7 confirmed by molecular diagnosis.

## 2 MATERIALS AND METHODS

### 2.1 First genetic test

After signing the consent form, blood samples were collected from the patient and her parents. Parent‐offspring trio whole‐exome sequencing was used to identify the causal variants. Sanger sequencing was performed as a confirmatory experiment. The reference sequence used was *SLC26A3* (NM_000111.3). According to the guidelines issued by the American College of Medical Genetics and Genomics (ACMG) in 2015, the pathogenicity of detected variations was determined.

### 2.2 Second genetic test

DNA was extracted from the individual and her parents’ peripheral blood. The genomic DNA of all three people was fragmented to produce 300–500 bp insert fragments. Custom‐designed NimbleGen SeqCap probes (Roche NimbleGen) were used for in‐solution hybridization to enrich target sequences, which included coding exons for about 5000 clinically relevant disease‐causing genes. Clinical‐Exome Sequencing sequenced on a NextSeq500 sequencer (Illumina) with 100–150 cycles of single‐end reads, according to the manufacturer's protocols. Besides detection of deleterious mutations and novel single nucleotide variants, coverage‐based algorithm developed in‐house, eCNVscan, was used to detect large exonic deletions and duplications. Sequencing reads were mapped to the reference of human genome version hg19 (2009–02 release, http://genome.ucsc.edu/).

## 3 RESULTS

### 3.1 Clinical findings

**FIGURE 1:**
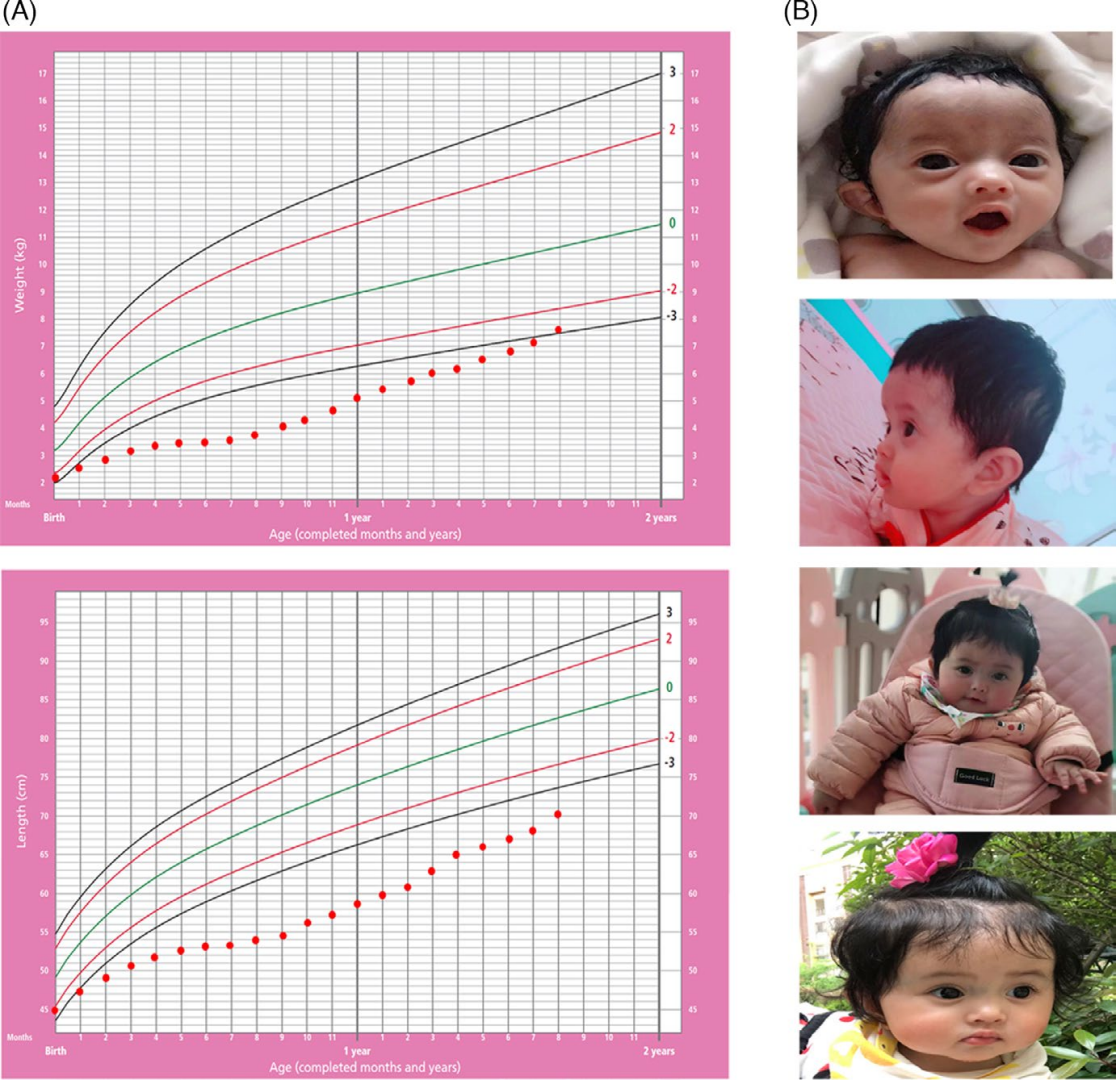
Clinical findings of the patient diagnosed with CCD combined with SRS. (A) Growth and weight charts. (B) Photographs of the patient at different months with triangular facial appearance and protruding forehead

The propositus was born as the first child of unrelated parents. She was delivered preterm via spontaneous vaginal delivery at 36^+5^ weeks (weight 2250 g, height 45 cm) (3%–10% and 10%–50%, respectively) after a hydramniotic pregnancy, and had no family history. She passed greenish watery stool 6–7 times per day without blood or mucous after birth. Later she was admitted to a local hospital at 3 month and diagnosed with Bartter syndrome characterized by metabolic alkalosis, hypochloremia, hypokalemia, and hyponatremia. However, gene testing indicated there was a homozygous mutation in the *SLC26A3* gene, which supported CCD diagnosis. Therefore, she received fluid replacement treatment to correct electrolytes and acid‐base disorder, followed by daily oral sodium chloride, potassium chloride, and indomethacin. The patient was presented to our hospital at 8 months of age with failure to thrive and watery diarrhea. She weighed 3.75 kg (−4.92 SD) and was 54.1 cm in length (−6.3 SD). On physical examination, she showed a protruding forehead, triangular facial appearance, and micrognathia (Figure 1B). Due to her short stature and specific facial appearance, we performed a second genetic test. The report showed segmental maternal UPD of chromosome 7q, so we revised the diagnosis to CCD combined with SRS. After regular follow‐up in our hospital, her diarrhea symptoms improved and lab tests (including serum electrolytes, blood gas analysis, serum glucose, IGF1, and IGFBP3) were almost normal. Her height and weight also increased, but her height was still lower than −3 SD (Figure 1A). We can just speculate that there are a certain cumulative clinical symptoms between these two defects. When we correct one of them, the clinical symptoms can be alleviated to a certain extent. But we are not 100% sure this phenomena is suitable for other patients because this is just one patient.

### 3.2 Genetic findings

**FIGURE 2:**
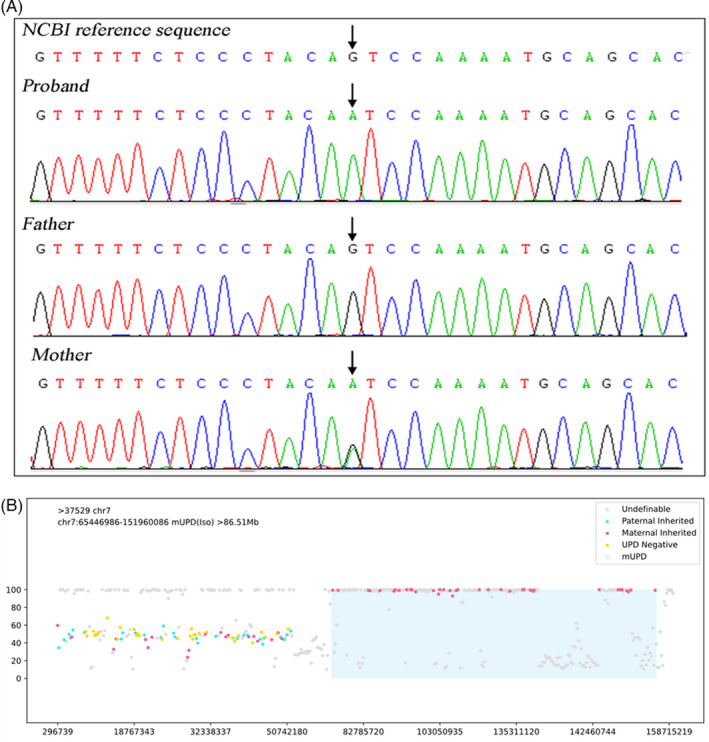
Genetic test results. (A) Pedigree and SLC26A3 variant identified in the family. The proband was the only patient in the family, and harbored a SLC26A3 variant. Mother of the patient was heterozygous whereas father of the patient was wild type. (B) SNP sites sequenced on chr7 (screened by sequencing quality) indicated that at least 86.51 Mb of genome 7q11q36 (chr7: 65446986–151960086) was maternal uniparental disomy

For the first genetic test, homozygous mutation of classical splicing site c.1515–1 G>A was found in the *SLC26A3* gene of the patient. The heterozygous mutation of this site was found in her mother, whereas her father was wild type (Figure 2A). However, the variant was not found in the ExAC (http://exac.broadinstitute.org) or HGMD (http://www.hgmd.cf.ac.uk) database. We interpreted NM_000111.3:c.1515‐1G>A as pathogenic according to the ACMG criteria with evidence levels PVS1+ PM2+PP4.

The second analysis showed that at least 86.51 Mb of genome 7q11q36 (chr7: 65446986–151960086) was loss of heterozygosity (AOH), and the sequencing data showed that the AOH in this chromosome interval occurred on the maternal chromosome, which was maternal haploid (mUPD‐iso) (Figure 2B). Sequencing data showed that the UPD may be novel; meanwhile, homozygous mutation c.1515‐1G>A in the *SLC26A3* gene (chr7:107417152) was detected in this mUPD‐iso region. Pathogenic CNVs were not identified in this patient.

## 4 DISCUSSION

In this study, the patient was first misdiagnosed with Bartter syndrome according to her clinical history and characteristic metabolic abnormalities. However, CCD diagnosis was determined by *SLC26A3* gene mutation. Later we found segmental maternal UPD of chromosome 7q, which included a genetic mutation in the *SLC26A3* gene. To the best of our knowledge, the present case is the first patient diagnosed with CCD combined with SRS caused by maternal UPD mutations including *SLC26A3* gene mutation.

Because CCD is very rare, especially in the Asian population, and clinical features of CCD patients frequently overlap with other conditions such as Bartter syndrome/Gitelman syndrome, when watery diarrhea is not observed or misinterpreted to be urine in early infancy, early identification and diagnosis are challenging. Clinical manifestation as well as urinary and stool chloride concentration is helpful in the differentiation of these diseases. However, mutation analysis of the *SLC26A3* gene may be useful to establish the diagnosis of CCD if clinical diagnosis is uncertain.

Early diagnosis and sufficient salt substitution therapy with potassium chloride and sodium chloride are the cornerstone of management and allow normal growth and development.^4^ Reduced severity of diarrheal episodes and diarrhea‐associated infant mortality have been observed in patients supplemented with vitamin A.^5^ Proton pump inhibitors (PPIs) namely omeprazole, effectively reduce the severity of CCD in certain cases.^6^ Butyrate, a short‐chain fatty acid, has pronounced effects on reducing the amount of diarrhea.^7^ However, even the same *SLC26A3* genotype can show different responses to oral butyrate therapy.^8^ Our patient was diagnosed at 3 months old by gene detection, and treated with electrolyte supply to correct the biochemical disorders. She is currently 20 months old, and her clinical symptoms have improved. She still needs regular follow‐up to prevent long‐term complications such as renal dysfunction and hyperuricemia.

SRS is currently clinically diagnosed based on a combination of characteristic features. However, patients with UPD(7)mat have less typical clinical features than those with 11p15 LOM,^9^ such as the patient in this study who had postnatal short stature, prominent forehead, and triangular face. Myoclonus dystonia, described in some children with UPD(7)mat, is likely related to the abnormal expression of sarcoglycan epsilon (SGCE) on chromosome 7q21.^10^, ^11^ Verbal dyspraxia and more global developmental retardation or learning impairment, usually not serious, occur in some cases with UPD(7)mat.^12^ UPD(7)mat patients have an increased prevalence of autistic spectrum disorder and lower cognitive ability (maybe caused by reduced brain volume of gray matter) compared to 11p15 LOM.^2^, ^13^ In SRS, children with UPD(7)mat seem to progress to puberty at an even younger age than patients with 11p15 LOM.^14^

Nutrition support, preventing hypoglycemia, and recovery of caloric‐related height or length defects are the main treatment goals of SRS patients in the first 2 years of life. In the consensus guidelines of 2017,^2^ a combination of GH and gonadotrophin‐releasing hormone analogs are recommended to improve height gain. Smeets et al.^15^ recently described that no metabolic differences were found before, during, and after GH treatment between SRS and non‐SRS patients born SGA. However, in a study by Lokulo‐Sodipe et al.,^16^ although ~70% of patients received GH treatment, their height still remained restricted (−3.13 SD), which means that past treatment regimens did not improve the height of adults with SRS. For the case in this paper, multi‐disciplinary treatment was administered by the nutrition department and rehabilitation department. The height and weight of the patient improved but were still lower than −2 SD. This may be partly due to the fact that the absorption function of the gastrointestinal tract caused by CCD did not completely improve, and secondly, it has a certain correlation with SRS. No language, motor, or intellectual issues were identified to date. We will continue tracking the growth and development of the child and will consider the use of GH treatment.

CCD and SRS are both rare diseases. A multi‐disciplinary approach is needed as well as close parental guidance. More research is urgently needed to prove basis investigation and more targeted management of patients with SRS combined with CCD.

## CONFLICT OF INTEREST

The authors have no conflicts of interest to declare.

## Data Availability

The data that support this study are included within the report.
